# A Simple and Objective Method for Reproducible Resting State Network (RSN) Detection in fMRI

**DOI:** 10.1371/journal.pone.0027594

**Published:** 2011-12-12

**Authors:** Gautam V. Pendse, David Borsook, Lino Becerra

**Affiliations:** 1 P.A.I.N Group, Imaging and Analysis Group (IMAG), McLean Hospital, Harvard Medical School, Belmont, Massachusetts, United States of America; 2 A. A. Martinos Center for Biomedical Imaging, Massachusetts General Hospital, Charlestown, Massachusetts, United States of America; University of California San Francisco, United States of America

## Abstract

Spatial Independent Component Analysis (ICA) decomposes the time by space functional MRI (fMRI) matrix into a set of 1-D basis time courses and their associated 3-D spatial maps that are optimized for mutual independence. When applied to resting state fMRI (rsfMRI), ICA produces several spatial independent components (ICs) that seem to have biological relevance - the so-called resting state networks (RSNs). The ICA problem is well posed when the true data generating process follows a linear mixture of ICs model in terms of the identifiability of the mixing matrix. However, the contrast function used for promoting mutual independence in ICA is dependent on the finite amount of observed data and is potentially non-convex with multiple local minima. Hence, each run of ICA could produce potentially different IC estimates even for the same data. One technique to deal with this run-to-run variability of ICA was proposed by [1] in their algorithm RAICAR which allows for the selection of only those ICs that have a high run-to-run reproducibility. We propose an enhancement to the original RAICAR algorithm that enables us to assign reproducibility 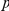-values to each IC and allows for an objective assessment of both within subject and across subjects reproducibility. We call the resulting algorithm RAICAR-N (N stands for null hypothesis test), and we have applied it to publicly available human rsfMRI data (http://www.nitrc.org). Our reproducibility analyses indicated that many of the published RSNs in rsfMRI literature are highly reproducible. However, we found several other RSNs that are highly reproducible but not frequently listed in the literature.

## Introduction

Independent component analysis (ICA) [2]–[5] models the observed data as a linear combination of a set of statistically independent and unobservable sources [6]. first proposed the application of ICA to the analysis of functional magnetic resonance imaging (fMRI) data. Subsequently, ICA has been applied to fMRI both as an exploratory tool for the purpose of identifying task related components [6] as well as a signal clean up tool for the purpose of removing artifacts from the fMRI data [7]. Recently, it has been shown that ICA applied to resting state fMRI (rsfMRI) in healthy subjects reveals a set of biologically meaningful spatial maps of independent components (ICs) that are consistent across subjects - the so called resting state networks (RSNs) [8]. Hence, there is a considerable interest in applying ICA to rsfMRI data in order to define the set of RSNs that characterize a particular group of human subjects, a disease, or a pharmacological effect.

Several variants of the linear ICA model have been applied to fMRI data including square ICA (with equal number of sources and sensors) [9], non-square ICA (with more sensors than sources) [6], and non-square ICA with additive Gaussian noise (noisy ICA) [10]. All of these models are well known in the ICA literature [2], [3], [5], [11]. Since the other ICA models are specializations of the noisy ICA model, we will assume a noisy ICA model henceforth.

Remarkably, the ICA estimation problem is well posed in terms of the identifiability of the mixing matrix given several non-Gaussian and at most 1 Gaussian source in the overall linear mixture [3], [12]–[14]. In the presence of more than 1 Gaussian source, such as in noisy ICA, the mixing matrix corresponding to the non-Gaussian part of the linear mixture is identifiable (upto permutation and scaling). In addition, the source distributions are uniquely identifiable (upto permutation and scaling) given a noisy ICA model with a particular Gaussian co-variance structure, for example, the isotropic diagonal co-variance. For details, see section 2.1.2.

While these uniqueness results are reassuring, a number of practical difficulties prevent the reliable estimation of ICs on real data. These difficulties include (1) true data not describable by an ICA model, (2) ICA contrast function approximations, (3) multiple local minima in the ICA contrast function, (4) confounding Gaussian noise and (5) model order overestimation. See section 2.1.3 for more details. A consequence of these difficulties is that multiple ICA runs on the same data or different subsets of the data produce different estimates of the IC realizations.

One technique to account for this run-to-run variability in ICA was proposed by [15] in their algorithm ICASSO. Using repeated runs of ICA with bootstrapped data using various initial conditions, ICASSO clusters ICs across ICA runs using agglomerative hierarchical clustering and also helps in visualizing the estimated ICs. The logic is that reliable ICs will show up in almost all ICA runs and thus will form a tight cluster well separated from the rest. [16] proposed a technique similar to ICASSO called self-organizing group ICA (sogICA) which allows for clustering of ICs via hierarchical clustering in across subject ICA runs. When applied to multiple ICA runs across subjects, ICASSO does not restrict the IC clusters to contain only 1 IC from each subject per ICA run. In contrast, sogICA allows the user to select the minimum number of subjects for a “group representative” IC cluster containing distinct subjects. By labelling each ICA run as a different “subject” sogICA can also be applied to analyze multiple ICA runs across subjects.

Similar in spirit to ICASSO and sogICA [1], proposed an intuitive approach called RAICAR (Ranking and Averaging Independent Component Analysis by Reproducibility) for reproducibility analysis of estimated ICs. The basic idea in RAICAR is to select only those ICs as “interesting” or “stable” which show a high run-to-run “reproducibility”. RAICAR uses simple and automated spatial cross-correlation matrix based IC alignment, which has been shown to be more accurate compared to ICASSO [1]. RAICAR is applicable to both within subject as well as across subjects reproducibility analysis.

A few limitations of ICASSO, sogICA and RAICAR are worth noting:

- ICASSO requires the user to select the number of IC clusters and is inapplicable without modification for across subjects analysis of ICA runs since the IC clusters are not restricted to contain only 1 IC per ICA run.
- sogICA requires the user to select the minimum number of subjects for a “group representative” cluster and also a cutoff on within cluster distances.
- RAICAR uses an arbitrary threshold on the reproducibility indices selected “by eye” or set at an arbitrary value, such as 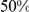 of the maximum reproducibility value.

We propose a simple extension to RAICAR that avoids subjective user decisions and allows for an automatic reproducibility cutoff. The reproducibility indices calculated in RAICAR differ in magnitude significantly depending on whether the input to RAICAR:

- (a) is generated using multiple ICA runs on the same data
- (b) comes from multiple ICA runs on varying data sets (e.g. between and across subject runs)

**Figure 1:**
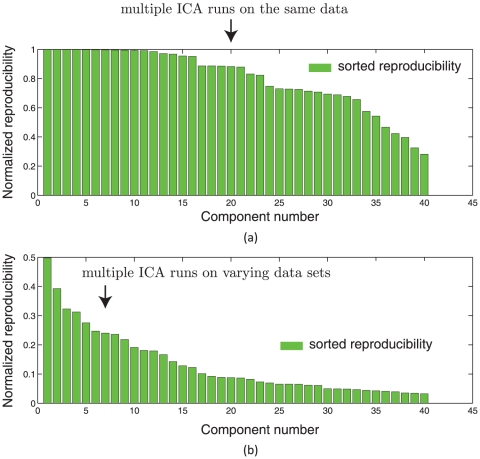
Figure illustrates the variation in normalized reproducibility from RAICAR depending on whether the input to RAICAR is (a) Multiple ICA runs on single subject data or (b) Multiple ICA runs across subjects. Notice that the normalized reproducibility is much lower for across subjects analysis compared to within subject analysis.

See Figure 1 for an illustration of this effect. Obviously, the reproducibility indices are much lower in case (b) since we account for both within subject and between subjects variability in estimating ICs. Case (b) is also of great interest from a practical point of view since we are often interested in making statements about a group of subjects. Hence, it is clear that a cutoff on RAICAR reproducibility values for the purposes of selecting the “highly reproducible” components should be data dependent. In this work,

1. We propose a modification of the original RAICAR algorithm by introducing an explicit “null” model of no reproducibility.
2. We use this “null” model to automatically generate 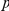-values for each IC via simulation. This allows for an objective cutoff specification for extracting reproducible ICs (e.g. reproducible at 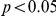) within and across subjects. We call the resulting algorithm RAICAR-N (N stands for “null” hypothesis test).
3. We validate RAICAR-N by applying it to publicly available human rsfMRI data.

### 1.1 Notation

- The set of real numbers will be denoted by 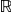. Scalars variables and functions will be denoted in a non-bold font (e.g., 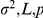 or 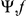). Vectors will be denoted in a bold font (except Greek letters) using lower case letters (e.g., 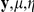). Matrices will be denoted in bold font using upper case letters (e.g., 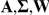). The transpose of a matrix 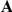 will be denoted by 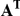 and its inverse will be denoted by 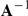. 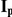 will denote the 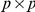 identity matrix and 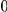 will denote a vector or matrix of all zeros whose size should be clear from context. 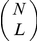 is the number of ways of choosing 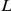 objects from 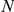 objects when order does not matter.
- The 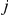
th component of vector 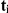 will be denoted by 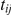 whereas the 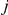
th component of vector 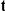 will be denoted by 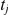. The element 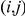 of matrix 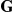 will be denoted by 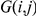 or 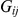. Estimates of variables will be denoted by putting a hat on top of the variable symbol. For example, an estimate of 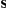 will be denoted by 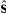.
- If 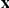 is a random vector with a multivariate Normal distribution with mean 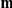 and covariance 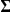 then we will denote this distribution by 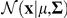. The joint density of vector 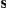 will be denoted by 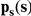 whereas the marginal density of 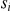 will be denoted as 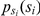. 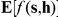 denotes the expectation of 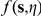 with respect to both random variables 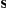 and 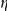.

## Methods

The organization of this article revolves around the following sequence of questions, which ultimately lead to the development of RAICAR-N:

1. Why is a reproducibility assessment necessary in ICA analysis? In order to answer this question, we cover the fundamentals of ICA including identifiability issues in sections 2.1 and 2.2.
2. How does the original RAICAR algorithm assess reproducibility? The answer to this question in section 2.3 will set up the stage for RAICAR-N.
3. How does RAICAR-N permit calculation of reproducibility 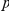-values? In section 2.4, we describe the RAICAR-N “null” model and a simulation based approach for assigning 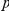-values to ICs.
4. How to promote diversity in group ICA runs given a limited number of subjects when using RAICAR-N and how to display the non-Gaussian spatial structure in estimated ICs? These issues are covered in section 2.5 and 2.6.
5. How can RAICAR-N be extended for between group comparison of ICs and how does it compare to other approaches in the literature? This question is addressed in section 4.4.

### 2.1 ICA background

In this section, we provide a brief introduction to ICA along with a discussion of associated issues related to model order selection, identifiability and run-to-run variability. The noisy ICA model assumes that observed data 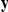 is generated as a linear combination of unobservable independent sources confounded with Gaussian noise:
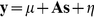
(2.1)


In this model,
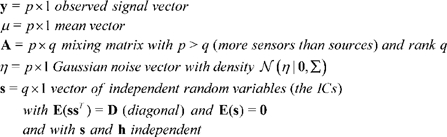
(2.2)If the marginal density of the 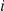
th source 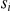 is 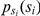 then the joint source density 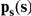 factorizes as 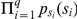 because of the independence assumption but is otherwise assumed to be unknown. Also, since the elements of 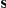 are independent their co-variance matrix 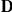 is diagonal. The set of variables 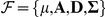 represents the unknown parameters in the noisy ICA model. Before discussing the identifiability of model 2.1, we briefly discuss the choice of model order or the assumed number of ICs 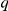.

#### 2.1.1 Estimating the model order 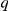


Rigorous estimation of the model order 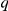 in noisy ICA is difficult as the IC densities 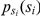 are unknown. This means that 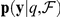, the marginal density of the observed data given the model order and the ICA parameters cannot be derived in closed form (by integrating out the ICs) without making additional assumptions on the form of IC densities. Consequently, standard model selection criteria such as Bayes information criterion (BIC) [17] cannot be easily applied to the noisy ICA model to estimate 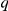. One solution is to use a factorial mixture of Gaussians (MOG) joint source density model as in [5], and use the analytical expression for 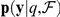 in conjunction with BIC. This solution is quite general in terms of allowing for an arbitrary Gaussian noise co-variance 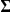, but maximizing 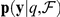 with respect to 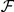 becomes computationally intractable using an expectation maximization (EM) algorithm for 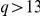 ICs [5]. Another rigorous non-parametric approach for estimating 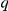 that is applicable to the noisy ICA model with isotropic diagonal Gaussian noise co-variance i.e., with 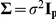 is the random matrix theory based sequential hypothesis testing approach of [18]. To the best of our knowledge, these are the only 2 rigorous approaches for estimating 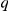 in the noisy ICA model.

Approximate approaches for estimating 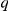 commonly used in fMRI literature (e.g., [10]) consist of first relaxing the isotropic diagonal noisy ICA model (with 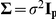) into a probabilistic PCA (PPCA) model of [19] where the source densities are assumed to be Gaussian i.e., where 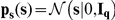. When using the PPCA model, it becomes possible to integrate out the Gaussian sources to get an expression for 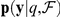 that can be analytically maximized [19]. Subsequently, methods such as BIC can be applied to estimate 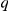. Alternative approaches for estimating 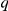 in the PPCA model consist of the Bayesian model selection of [20], or in data-rich situations such as fMRI, even the standard technique of cross-validation [21].

From a biological point of view, it has been argued [22] that the number of extracted ICs simply reflect the various *equally valid* views of the human functional neurobiology - smaller number of ICs represent a coarse view while a larger number of ICs represent a more fine grained view. However, it is worth noting that from a statistical point of view, over-specification of 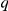 will lead to over-fitting of the ICA model, which might render the estimated ICs less generalizable across subjects. On the other hand, under-specification of 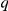 will result in incomplete IC separation. Both of these scenarios are undesirable.

#### 2.1.2 Identifiability of the noisy ICA model

To what extent is the noisy linear ICA model identifiable? Consider a potentially different decomposition of the noisy ICA model 2.1:
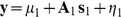
(2.3)where
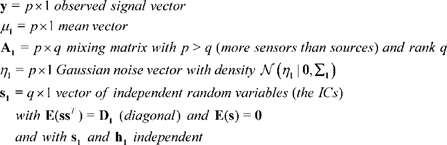
(2.4)What can be said about the equivalence between the parameterizations in 2.1 and 2.3?

*Identifiability of* 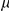: Equating the expectations of the right hand size of 2.3 and 2.1 and noting that 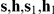 have mean 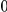 we get:
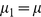
(2.5)Thus the mean vector 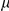 is exactly identifiable.

*Identifiability of* 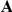 *:* A fundamental decomposition result states that the noisy ICA problem is well-posed in terms of the identifiability of the mixing matrix 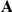 upto permutation and scaling provided that the components of 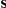 are independent and **non-Gaussian** [3], [12]–[14]. If 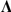 is a diagonal scaling matrix and 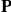 is a permutation matrix then the identifiability result can be stated as:
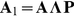
(2.6)where 2.3 is another decomposition of 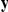 with 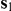 containing independent and non-Gaussian components. In other words, the mixing matrix 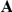 is identifiable upto permutation and scaling.

*Identifiability of* 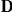 *and* 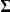 *:* Equating the second moments of the right hand side of 2.3 and 2.1 and noting the equality of means 2.5 and the independence of 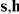 and 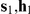 we get:
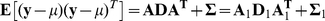
(2.7)


Let 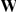 be a 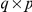 matrix and 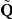 be a 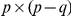 orthogonal matrix such that:
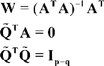
(2.8)From 2.8 and 2.7 we get:
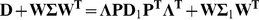
(2.9)

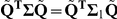



Case 1: 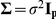 and 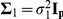. The second equation in 2.9 along with the orthogonality of 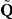 gives 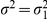 and thus 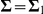. If we fix the scaling of 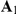 by selecting 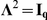 then from the first equation in 2.9 we get:
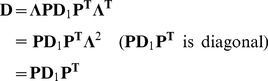
(2.10)In other words, the noise co-variance 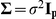 is uniquely determined and for a fixed scaling 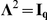, the source variances 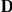 are also uniquely determined upto permutation.

Case 2: 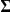 and 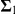 arbitrary positive definite matrices. Suppose 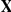 is a square matrix and let 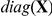 be the diagonal matrix obtained by setting the non-diagonal elements of 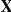 to 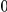 and similarly let 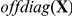 be the matrix obtained by setting the diagonal elements of 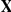 to 0. The noise-covariance is partially identifiable by the following conditions:
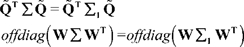
(2.11)For a fixed scaling 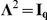, the sources variances 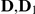 are constrained by:
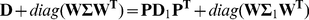
(2.12)


In general, the source variances 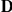 cannot be uniquely determined as noted in [14].

*Identifiability of the distribution of* 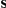 *:* Is the distribution of the non-Gaussian components of 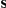 identifiable? From 2.1 and 2.3:
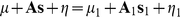
(2.13)Substituting 2.5 and 2.6 in 2.13 we get:
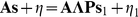
(2.14)Left multiplying both sides by 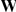 from 2.8 we get:
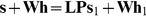
(2.15)Let 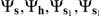 be the characteristic functions of 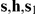 and 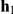 respectively. Then
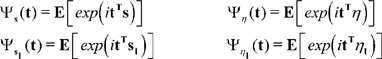
(2.16)where 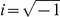 and 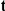 is a vector of real numbers of length equal to that of the corresponding random vectors in 2.16. Using 2.15, we can write:
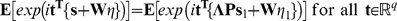
(2.17)Noting the independence of 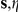 and 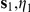:
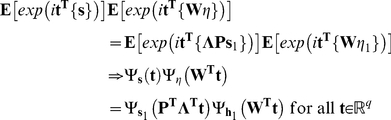
(2.18)Now 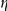 and 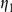 are multivariate Gaussian random vectors both with mean 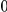 and co-variance matrix 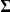 and 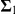 respectively. Hence, their characteristic functions are given by [23], [24]:
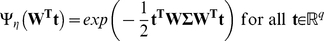
(2.19)

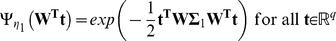



**Claim 2.1** *A sufficient condition for identifiability upto permutation and scaling of the non-Gaussian distributions in* 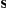 *given two different parameterizations in 2.1 and 2.3 is:*

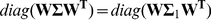
(2.20)


**Proof**• From 2.20 and 2.11, we get:
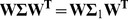
(2.21)Thus from 2.19,
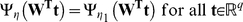
(2.22)From 2.19, 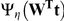 and 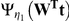 are not equal to 0 for any finite 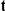, therefore, from 2.22 and 2.18 we get:
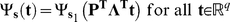
(2.23)Note that 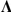 is a diagonal scaling matrix with entries 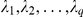 on the diagonal and 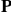 is a permutation matrix. Thus,
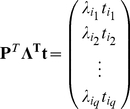
(2.24)where 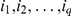 is some permutation of integers 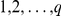. Suppose 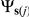 is the characteristic function of the 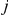
th component of 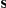 and 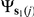 is the characteristic function of the 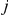
th component of 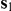. Since the components of 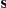 and 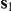 are independent by assumption, the joint characteristic functions 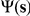 and 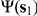 factorize:
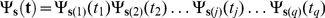
(2.25)

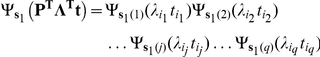
From 2.25 and 2.23
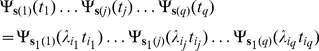
(2.26)


All characteristic functions satisfy [23], [24]:
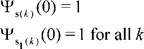
(2.27)


Since 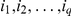 is simply a permutation of integers 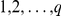, there exists a 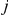 such that 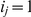. Then set 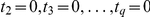 in 2.26. Then 2.27 and 2.26 imply:
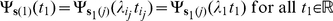
(2.28)


Select the scaling matrix as 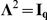 and thus 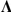 is a diagonal matrix with elements 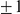 on the diagonal. Thus 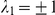 and 2.28 can be re-written as:
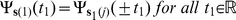
(2.29)Therefore,
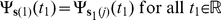
(2.30)

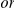


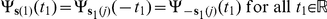



Hence the characteristic function of the 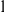
st component of 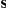 is identical to the characteristic function of the (possibly sign-flipped) 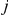
th component of 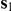. Since characteristic functions uniquely characterize a probability distribution [23], the distribution of 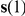 and 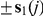 is identical. Next, by setting 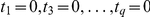, we can find a distribution from 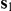 that matches the 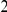
nd component 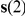 of 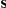. Proceeding in a similar fashion, it is clear that the distribution of each component of 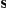 is uniquely identifiable upto sign flips for the choice 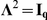. For a general 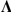, the source distributions are uniquely identifiable upto permutation and (possibly negative) scaling, as claimed.

While the source distributions might not be uniquely identifiable for arbitrary co-variance matrices 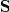, they are indeed uniquely identifiable upto permutation and scaling for the noisy ICA model with isotropic Gaussian noise co-variance. For more general conditions that guarantee uniqueness of source distributions, please see [25], [26].

**Corollary 2.2** *If* 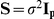 *and* 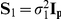, *then the source distributions are uniquely identifiable upto sign flips for* 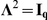.

**Proof** Suppose 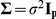 and 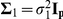. Then from 2.9 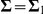 and thus 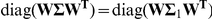. The corollary then follows from Claim 2.1.

**Corollary 2.3** *If* 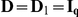, *then the source distributions are uniquely identifiable up to sign flips for* 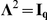.

**Proof** If 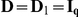, then noting that 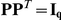, we get 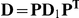. Hence from 2.12, we get 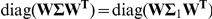. The corollary then follows from Claim 2.1.

#### 2.1.3 Why is there a run-to-run variability in estimated ICs?

From the discussion in section 2.1.2, it is clear that for a noisy ICA model with isotropic diagonal additive Gaussian noise co-variance:

1. The noisy ICA parameters 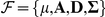 are uniquely identifiable up to permutation and scaling.
2. The source distributions in 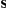 are uniquely identifiable upto permutation and scaling.

While the above theoretical properties of ICA are reassuring, there are a number of practical difficulties that prevent the reliable estimation of ICs on real data:

1. *Validity of the ICA model:* The assumption that the observed real data is generated by an ICA model is only that - an “assumption”. If this assumption is not valid, then the uniqueness results do not hold anymore.
2. *Mutual information approximations:* From an information theoretic point of view, the ICA problem is solved by minimizing a contrast function which is an *approximation* to the mutual information [27] between the ICs that depends on the finite amount of observed data. Such an approximation is necessary, since we do not have access to the marginal source densities 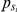. Different approximations to mutual information will lead to different objective functions and hence different solutions. This is one of the reasons why different ICA algorithms often produce different IC estimates even for the same data.
3. *Non-convexity of ICA objective functions:* The ICA contrast function is potentially non-convex and hence has multiple local minima. Since global minimization is a challenging problem by itself, most ICA algorithms will only converge to local minima of the ICA contrast function. The run-to-run variability of IC estimates will also depend on the number of local minima in a particular ICA contrast function.
4. *IC estimate corruption by Gaussian noise:* For noisy ICA, the IC realizations cannot be recovered exactly even if the true mixing matrix 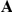 and mean vector 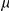 are known in 2.1. Commonly used estimators for recovering realization of ICs include the least squares [10] as well as the minimum mean square error (MMSE) [14]. Consider the least squares estimate 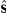 of a realization of 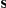 based on 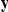:
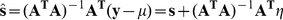
(2.31)This means that even for known parameters, IC realization estimates 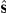 will be corrupted by correlated Gaussian noise. Hence using different subsets of the data under the true model will also lead to variability in estimated ICs.
5. *Over-fitting of the ICA model:* Over specification of the model order leads to the problem of over-fitting in ICA. As we describe below, this can lead to (1) the phenomenon of IC “splitting” and (2) an increase in the variance of the IC estimates.

*1. IC “splitting”*

Suppose that the true model order or the number of non-Gaussian sources in an ICA decomposition of 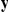 such as 2.1 is 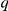. Then a fundamental result in [12, Theorem 1] states that for any other ICA decomposition of 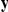, the number of non-Gaussian sources remains the same while the number of Gaussian sources can change. In other words, 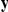 cannot have two different ICA decompositions containing different number of non-Gaussian sources.

In view of this fact, how can a model order 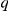 ICA decomposition containing 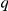 non-Gaussian sources be “split” into a 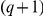 ICA decomposition containing 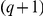 non-Gaussian sources when performing ICA estimation using an assumed model order of 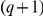
? As we describe below, the order 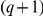 ICA decomposition is only an *approximation* to the order 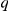 ICA decomposition.

Let 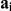 be the 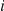
th column of 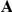 in 2.1. In the presence of noise, it might be possible to *approximate*:
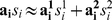
(2.32)Here:

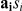
 is the contribution of the 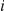
th non-Gaussian source 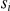 to the ICA model 2.1.

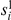
 and 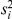 are independent non-Gaussian random variables that are also independent with respect to all non-Gaussian sources 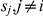 in 2.1.

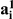
 and 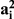 are the basis time courses corresponding to 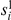 and 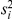 respectively.
- The time courses 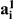 and 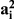 look similar to each other.

Note that if 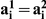, then 2.32 can be made into an equality by choosing 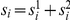. By replacing 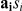 in 2.1 using 2.32, we arrive at an *approximate* model order 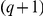 decomposition of 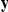. In this decomposition, the component 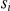 from a model order 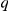 decomposition appears to be “split” into two sub-components: 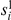 and 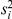.

*2. Inflated variance of IC estimates*

Overestimation of model order will lead to over-fitting of the mixing matrix 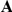. In other words, 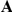 could have several columns that are highly correlated with each other. This could happen as a result of IC “splitting” as discussed above. Now, for a given realization 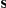, the variance of 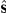 is given by 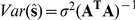 (for isotropic Gaussian co-variance). An increase in number of columns of 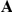 and the fact that many of them are highly correlated implies that the variability of IC estimates 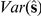 is inflated.

In other words, running ICA multiple times on the same data or variations thereof with random initialization could produce different ICs.

### 2.2 ICA algorithms, single subject ICA and group ICA

In this section, we give a brief summary of how the ICA parameters are estimated in practice and also summarize the two most common modes of ICA application to fMRI data - single subject ICA (section 2.2.1) and temporal concatenation based group ICA (section 2.2.2).

Given several independent observations 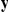 as per the noisy ICA model 2.1, most ICA algorithms estimate the ICA parameters 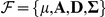 and the realizations of 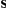 in 2 steps. We only consider the case with 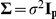, since as shown in section 2.1.2, the mixing matrix 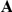 and source distributions of 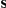 are identifiable upto permutation and scaling for this case.

1. First, the diagonal source co-variance is arbitrarily set as 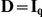. The mean vector 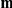 is estimated as 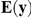. Then, using PCA or PPCA [19], the mixing matrix 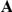 is estimated, upto an orthogonal rotation matrix 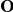, to be in a signal subspace which is spanned by the principal eigenvectors corresponding to the largest eigenvalues of the data co-variance matrix 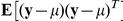. The noise variance 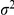 is estimated in this step as well.
2. Next, an estimator 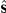 for the source realizations is defined using techniques such as least squares or MMSE. The only unknown involved in these estimates is the orthogonal rotation matrix 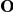.
3. Finally, the non-Gaussianity of the empirical density of components of 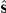 is optimized with respect to 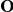 using algorithms such as fixed point ICA [27], [28].

For more details on noisy ICA estimation, please see [10] and for more details on ICA algorithms, please see [29].

#### 2.2.1 Single subject ICA

How is ICA applied to single subject fMRI data? Suppose we are given a single subject fMRI scan which we rearrange as a 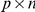 2D matrix 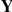 in which column 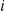 is the 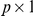 observed time-course 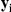 in the brain at voxel 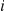. Observed time-courses 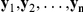 are considered to be 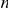 independent realizations of 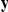 as per the linear ICA model 2.1. Suppose 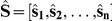 is the 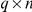 matrix containing the estimated source realizations at the 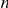 voxels. The 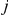 th row of 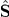 is the 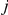
th IC. In other words, we decompose the time by space fMRI 2D matrix into a set of basis time-courses and a set of 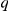 3D IC maps using ICA.

#### 2.2.2 Group ICA

How is ICA applied to data from a group of subjects in fMRI? Suppose we collect fMRI images from 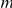 subjects. First, we register all subjects to a common space using a registration algorithm (e.g., affine registration). Next, we rearrange each of the fMRI scans into 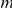 2D matrices 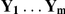, each of size 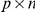. Column 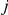 in 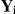 is the demeaned time-course observed at voxel location 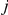 for subject 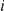. The matrices 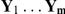 are temporally concatenated to get a 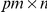 matrix 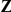 as follows:
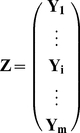
(2.33)Column 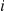 of 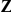 is the 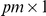 vector 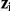 which is assumed to follow a linear ICA model 2.1. 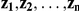 are considered to be independent realizations of the model 2.1. Suppose 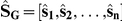 is a 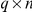 matrix containing the estimated source realizations at the 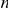 voxels. The 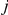
th row of 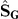 is the 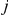 th group IC. In group ICA, the joined time-series across subjects is modeled using noisy linear ICA. In practice, 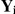 is the PCA reduced data set for subject 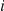. The PCA reduction is either done separately for each subject using subject specific data co-variance [9] or an average data co-variance across subjects [8]. The average co-variance approach requires each subject to have the same number of time points in fMRI scans.

### 2.3 The original RAICAR algorithm

**Figure 2:**
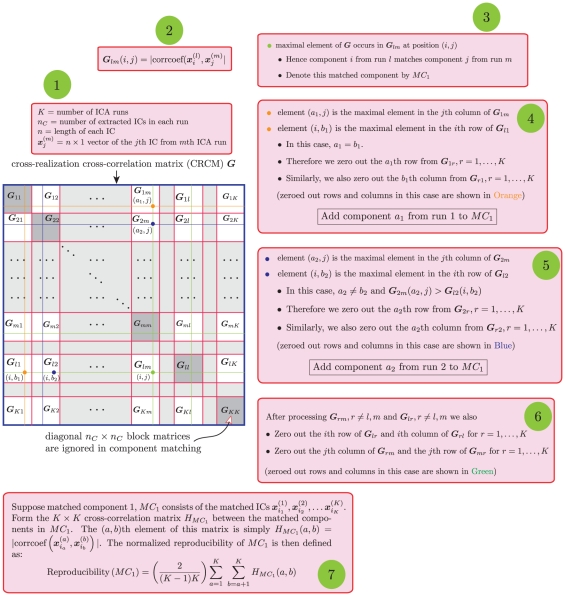
Pictorial depiction of the original RAICAR algorithm [**1**]. The ICA algorithm is run 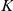 times with each run producing 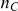 ICs. 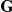 is a 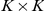 block matrix with elements 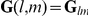 where 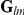 is the 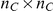 absolute spatial cross-correlation matrix between ICs from runs 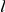 and 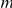. The numbered green circles indicate the sequence of steps in applying RAICAR to a given data set. Our definition of normalized reproducibility in box 7 averages un-thresholded correlation coefficients thereby avoiding the selection of a correlation coefficient threshold prior to averaging.

In this section, we give a brief introduction to the RAICAR algorithm of [1]. Suppose we are given a data set which we decompose into 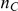 ICs using ICA (e.g., single subject or group ICA). Our goal is to assess which ICs consistently show up in multiple ICA runs i.e., the reproducibility of each of these 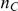 ICs. To that extent, we run the ICA algorithm 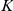 times. Suppose 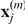 is the 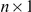 vector (e.g. spatial ICA map re-arranged into a vector) of the 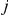 th IC from 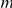 th ICA run. Suppose 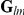 is a 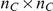 absolute spatial cross-correlation coefficient matrix between the ICs from runs 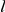 and 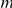:
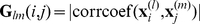
(2.34)where 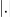 denotes absolute value. 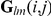 is the absolute spatial cross-correlation coefficient between IC 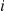 from run 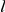 and IC 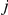 from run 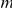. The matrices 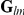 are then arranged as elements of a 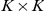 *block-matrix* 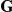 such that the 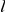 th row and 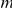 th column of 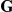 is 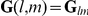 (Figure 2). This block matrix 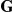 is the starting point for a RAICAR across-run component matching process.

Since ICs within a particular run cannot be matched to each other, the 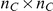 matrices 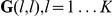 along the block-diagonal of 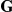 are set to 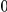 as shown in Figure 2 with a gray color. The following steps are involved in a RAICAR analysis:

1. Find the maximal element of 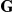. Suppose this maximum occurs in matrix 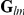 at position 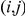. Hence component 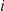 from run 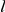 matches component 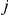 from run 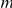. Let us label this matched component by 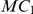 (the first matched component).
2. Next, we attempt to find from each run 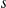 (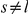 and 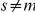) a component that matches with component 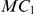. Suppose element 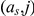 is the maximal element in the 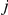 th column of 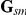. Then component 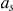 is the best matching component from run 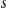 with the 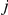 th component from run 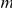.

Similarly, suppose element 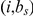 is the maximal element in the 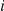 th row of 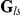. Then component 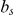 is the best matching component from run 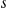 with component 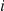 from run 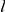. As noted in [1], in most cases 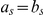. However, it is possible that 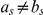. Hence the component number 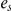 matching 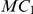 from run 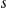 is defined as follows:
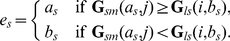
(2.35)


We would also like to remove component 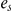 of run 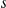 from further consideration during the matching process. To that extent, we zero out the 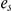 th row from 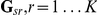 and the 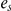 th column from 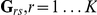.

1. Once a matching component 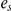 has been found for all runs 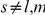, we also zero out the 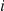
th row from 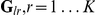 and the ith column from 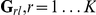. Similarly, we zero out the 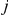 th column from 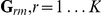 and the 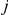 th row from 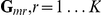. This eliminates component 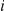 from run 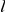 and component 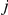 from run 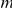 from further consideration during the matching process.
2. Steps 1–3 complete the matching process for one IC component across runs. These steps are repeated until 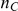 components are matched across the 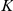 runs. We label the matched component 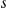 as 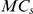 which contains a set of 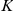 matching ICs one from each of the 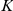 ICA runs.

Suppose matched component 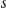, 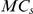 consists of the matched ICs 
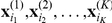
. Form the 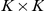 cross-correlation matrix 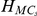 between the matched components in 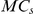. The 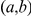 th element of this matrix is simply:
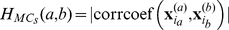
(2.36)The normalized reproducibility of 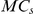 is then defined as:
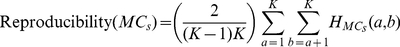
(2.37)


The double sum in 2.37 is simply the sum of the upper triangular part of 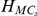 excluding the diagonal. The normalizing factor 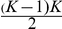 is simply the maximum possible value of this sum. Hence the normalized reproducibility satisfies: 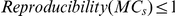.

Note that our definition of normalized reproducibility is slightly different from that in [1]. Whereas [1] averages the *thresholded* absolute correlation coefficients, we simply average the *un-thresholded* absolute correlation coefficients to compute reproducibility thereby avoiding the selection of a threshold on the absolute correlation coefficients.

### 2.4 The RAICAR-N enhancement

In this section, we describe how to compute reproducibility 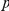-values for each matched component in RAICAR. Note that the RAICAR “component matching” process can be used to assess the reproducibility of *any* spatial component maps - not necessarily ICA maps. For instance, RAICAR can be used to assess the reproducibility of a set of PCA maps across subjects.

In order to generate reproducibility 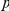-values for the matched component maps:

1. We need to determine the distribution of normalized reproducibility that we get from the RAICAR “component matching” process when the input to RAICAR represents a set of “non-reproducible component maps” across the 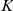 runs.
2. In addition, we would also like to preserve the *overall structure* seen in the *observed* sets of spatial component maps across the 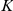 runs when generating sets of “non-reproducible component maps” across the 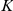 runs.

Hence for IC reproducibility assessment, we propose to use the original set of ICs across the 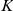 runs to generate the “non-reproducible component maps” across the 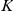 runs.

Suppose 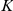 ICA runs are submitted to RAICAR which gives us a 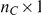 vector of *observed* normalized reproducibility values 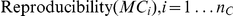 - one for each IC. We propose to attach 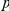-values for measuring the reproducibility of each IC in a data-driven fashion as follows:

1. First, we label the 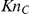 ICs across the 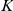 runs using unique integers. In run 1, the ICs are labelled using integers 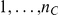. In run 2, the ICs are labelled using integers 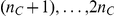 and so on. In run 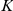, the ICs are labelled using integers 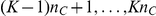.
2. Our “null” hypothesis is:
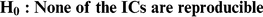
(2.38)

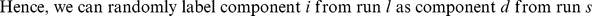

To do this, we randomly permute the integers 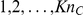 to get the permuted integers 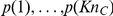. Obviously 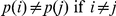.

1. The 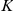 sets “non-reproducible component runs under 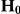
” are constructed by assigning components with labels:

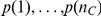
 to run 1 under 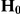.

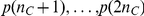
 to run 2 under 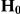

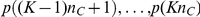
 to run 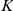 under 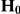

**Figure 3:**
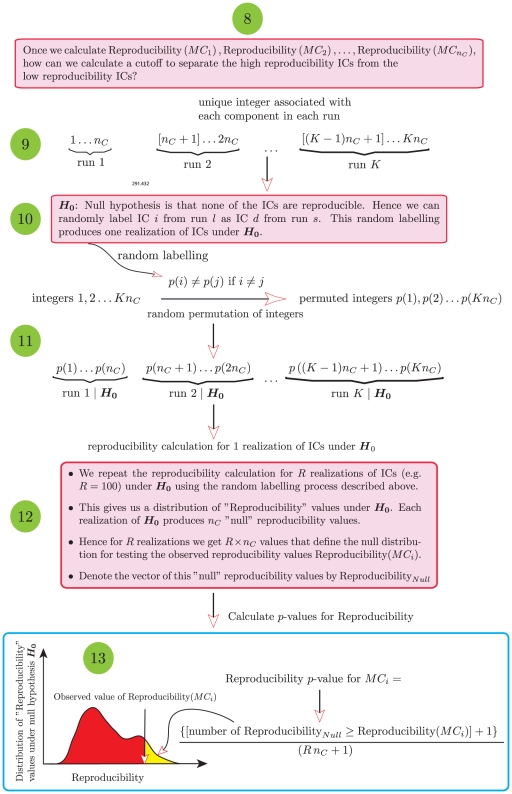
Pictorial depiction of the process for generating a “null” distribution in RAICAR-N. Our “null” hypothesis is: “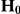: None of the ICs are reproducible. Hence, we can randomly label IC 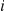 from run 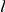 as IC 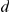 from run 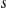
”. Therefore we randomly split the 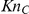 ICs across 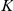 runs into 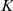 parts and run the RAICAR algorithm on each set of randomly split ICs. This gives us a set of “null” reproducibility values which can be used to compute 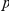-values for the observed reproducibility of ICs in the original RAICAR run. The green circles indicate the sequence of steps for generating the “null” distribution after the steps in Figure 2.

3. After 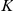 runs have been generated under 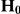, we subject these to a RAICAR analysis. This gives us 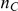 values of normalized reproducibility, one for each matched component under 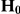.
4. Steps 1–4 are repeated 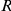 times to build up a *pooled* 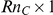 vector of normalized reproducibility 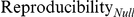 under 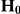.
5. Finally, we assign a 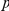-value for reproducibility to each matched IC across the 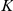 runs. The observed reproducibility for 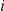 th matched IC is 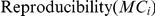 and its 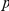-value is:
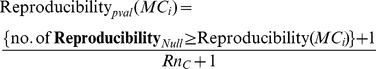
(2.39)
6. Only those components with 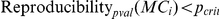 are considered to be significantly reproducible. We can use a fixed and objective value for 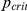 such as 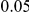. Note that this fixed cutoff is independent of the amount of variability in the input to RAICAR-N. Please see Figure 3 for a pictorial depiction of this process.

**Figure 4:**
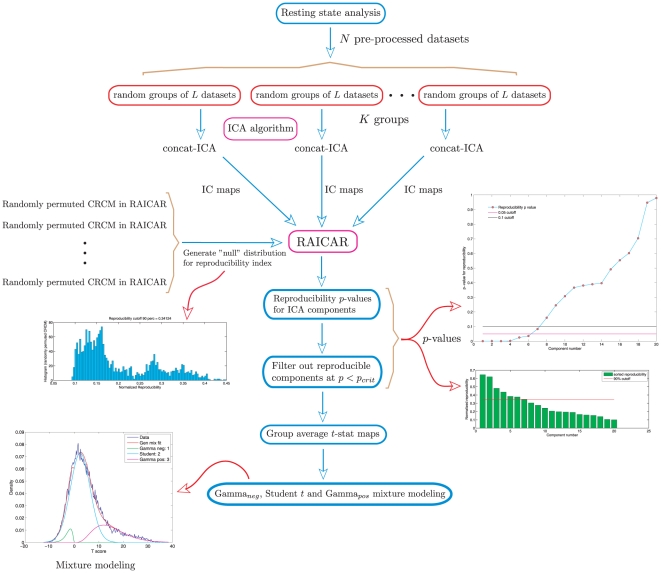
Flowchart for a group ICA based RAICAR-N analysis. The 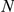 single subject data sets are first pre-processed and subsequently bootstrapped to create 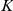 groups, each group containing 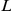 distinct subjects. Each group of 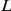 subjects is submitted to a temporal concatenation group ICA analysis. The resulting IC maps (either raw ICs or ICs scaled by noise standard deviation) are subjected to a RAICAR analysis. The cross-realization cross correlation matrix (CRCM) is randomly permuted multiple times: 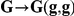 where 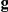 is a random permutation of integers from 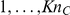. The permuted CRCMs are subjected to a RAICAR analysis to generate a realization of reproducibility values under the “null” hypothesis. The computed “null” distribution of reproducibility values is used to assign 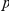 values to the observed reproducibility of the original RAICAR run. Finally, reproducible ICs are averaged using a random effects analysis and the resulting 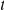-statistic images are subjected to Gamma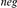, Student 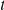 and Gamma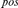 mixture modeling.

Figure 4 shows a schematic of the RAICAR-N analysis process.

### 2.5 How many subjects should be used per group ICA run in RAICAR-N?

The input to RAICAR-N can either be single subject ICA runs or group ICA runs across a set of subjects. Note that the individual subject ICA runs are spatially unconstrained whereas a group ICA spatially constrains the group ICs across a set of subjects. Hence the number of ICs that can be declared as significantly reproducible at the group level are usually more than those that can be declared significantly reproducible at the single subject level. Hence the following question is relevant:

Suppose we have a group of 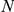 subjects. We randomly select 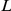 subjects and form a single group of subjects. We repeat this process 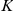 times to get 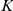 groups of 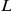 subjects each of which is subjected to a group ICA analysis. Given the number of subjects 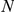, how should we choose 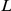 and 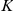
?

First, we discuss the choice of 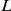. If 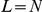 then each of the 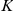 groups will contain the same 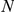 subjects and hence there will be no diversity in the 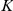 groups. We would like to control the amount of diversity in the 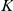 groups of 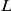 subjects. Consider any 2 subjects 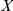 and 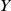. The probability 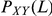 that both 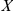 and 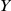 appear in a set of 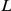 randomly chosen subjects from 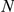 subjects is given by:
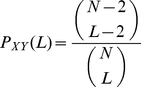
(2.40)


The expected number of times that 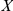 and 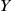 appear together in sets of 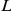 subjects out of 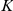 independently drawn sets is:
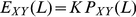
(2.41)


Ideally, we would like 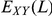 to be only a small fraction of 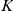. Hence we impose the restriction:
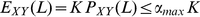
(2.42)where 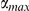 is a user defined constant such as 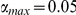. This implies that the chosen value of 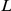 must satisfy:
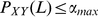
(2.43)


**Figure 5:**
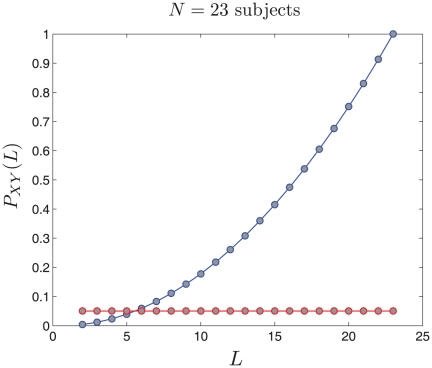
Figure shows a plot of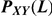 vs 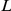 for 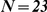 in blue. The red line shows the 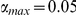 cutoff. The largest value of 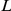 for which 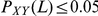 is 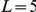.

In practice, we choose the largest value of 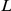 that satisfies this inequality. As shown in Figure 5, if 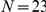 and 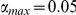 then the largest value of 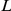 that satisfies 2.43 is 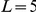. The number of group ICA runs 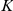 should be as large as possible. From our experiments on real fMRI data we can roughly say that values of 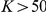 give equivalent results.

### 2.6 How to display the estimated non-Gaussian spatial structure in ICA maps?

The ICs have been optimized for non-Gaussianity. However, there can be many types of non-Gaussian distributions. It has been empirically found that the non-Gaussian distributions of ICs found in fMRI data have the following structure:

1. A central Gaussian looking part and
2. A tail that extends out on either end of the Gaussian

It has been suggested in [10] that a Gaussian/Gamma mixture model can be fitted to this distribution and the Gamma components can be thought of as representatives of the non-Gaussian structure. We follow a similar approach:

1. The output of a RAICAR-N analysis is a set of spatial ICA maps (either 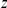-transformed maps or raw maps) concatenated into a 4-D volume.
2. We do a voxelwise transformation to Normality using the voxelwise empirical cumulative distribution function as described in [30].
3. Next, we submit the resulting 4-D volume to a voxelwise group analysis using ordinary least squares. The design matrix for group analysis depends on the question being considered. In our case, the design matrix was simply a single group average design.
4. The resulting 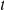-statistic maps are subjected to Student 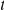, Gamma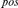 and Gamma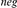 mixture modeling. The logic is that if the original ICA maps are pure Gaussian (i.e., have no interesting non-Gaussian structure) then the result of a group average analysis will be a pure Student 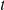 map which will be captured by a single Student 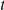 (i.e., the Gamma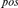 and Gamma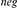 will be driven to 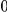 class fractions). Hence the “null” hypothesis will be correctly accounted for.
5. If the Gamma distributions have 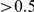 posterior probability at some voxels then those voxels are displayed in color to indicate the presence of significant non-Gaussian structure over and above the background Student 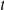 distribution.

**Figure 6:**
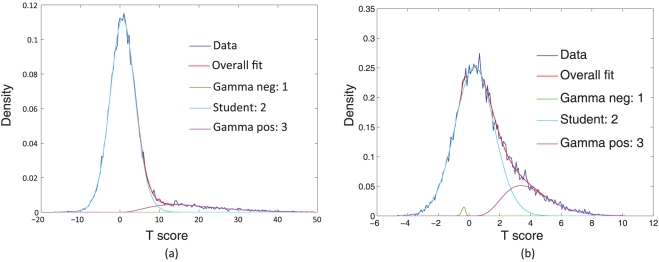
Examples of displaying non-Gaussian spatial structure using a Student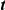, Gamma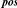 and Gamma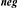 mixture model. Notice how the Gamma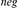 density is driven to near 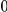 class fraction in the absence of significant negative non-Gaussian structure.

Examples of Student 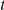, Gamma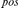 and Gamma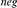 mixture model fits are shown in Figure 6.

## Results

### 3.1 Human rsfMRI data

rsfMRI data titled: Baltimore (Pekar, J.J./Mostofsky, S.H.; n = 23 [8M/15F]; ages: 20–40; TR = 2.5; # slices = 47; # timepoints = 123), a part of the 1000 functional connectomes project, was downloaded from the Neuroimaging Informatics Tools and Resources Clearinghouse (NITRC): http://www.nitrc.org/projects/fcon_1000/.

### 3.2 Preprocessing

**Figure 7:**
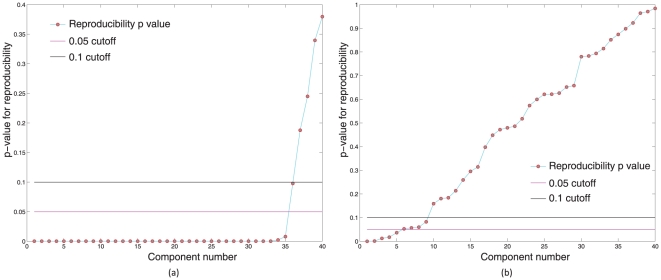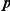-value cutoffs for within and across single subject analysis using RAICAR-N. This figure illustrates the intuitive fact that within subject ICA runs are much more reproducible compared to across subject ICA runs.

Data was analyzed using tools from the FMRIB software library (FSL: http://www.fmrib.ox.ac.uk/fsl/). Preprocessing steps included motion correction, brain extraction, spatial smoothing with an isotropic Gaussian kernel of 5 mm FWHM and 100 s high-pass temporal filtering. Spatial ICA was performed using a noisy ICA model as implemented in FSL MELODIC [10] in either single subject or multi-subject temporal concatenation mode also called group ICA. Please see section 2.2 for a brief summary of single subject ICA and group ICA. In each case, we fixed the model order of ICA at 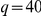 to be consistent with the model order range typically extracted in rsfMRI and fMRI [16], [31]. For temporal concatenation based group ICA, single subject data was first affinely registered to the MNI 152 brain and subsequently resampled to 4×4×4 resolution (MNI 4×4×4) to decrease computational load. Please see Figure 4 for a schematic of the RAICAR-N analysis process. In this work, we report across subject RAICAR-N analyses, but as shown in Figure 7, within subject ICA runs can also be entered into RAICAR-N.

### 3.3 RAICAR-N analysis with 1 ICA run per subject

**Figure 8:**
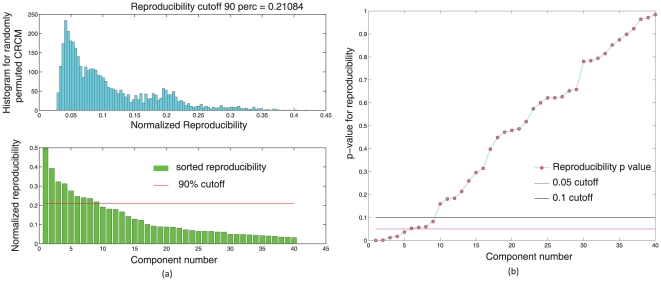
Single subject rsfMRI ICA runs across 23 subjects were combined using a RAICAR-N analysis. Figure (a) shows the observed values of normalized reproducibility (bottom) as well as the “null” distribution of normalized reproducibility across 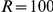 simulations (top). Figure (b) shows the 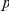-values for each IC along with the 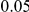 and 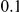 cutoff lines.

Spatial ICA was run once for each of the 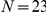 subjects in their native space. The resulting set of ICA components across subjects were transformed to MNI 4×4×4 space and were submitted to a RAICAR-N analysis. In all RAICAR-N analyses reported in this article, we used the 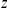-transformed IC maps - which are basically the raw IC maps divided by a voxelwise estimate of noise standard deviation (named as melodic_IC.nii.gz in MELODIC). It is also possible to use the raw IC maps as inputs to RAICAR-N. ICA components were sorted according to their reproducibility and 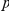-values were computed for each ICA component. Please see Figure 8.

**Figure 9:**
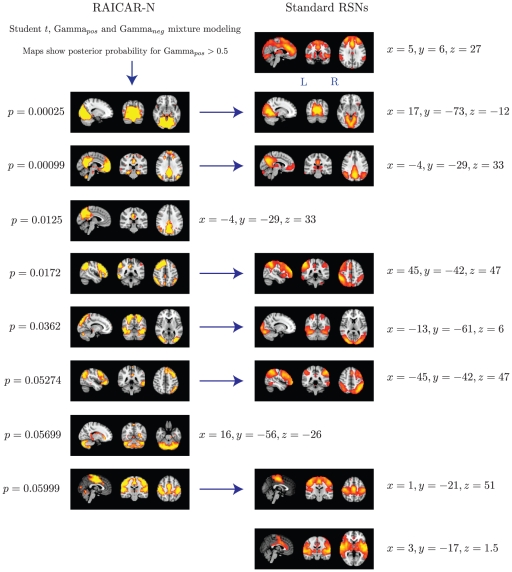
The top 8 “reproducible” ICs from a RAICAR-N analysis on single subject ICA runs compared with standard RSN maps reported in literature [8]. We are able to declare 4 “standard” RSNs as significantly reproducible at a 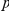-value 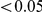. There are 2 other “standard” RSNs that achieve a reproducibility 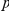-value between 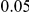 and 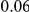 as well as 2 “non-standard” RSNs that achieve 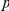-values of 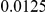 and 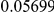 respectively. We also could not find 2 of the published RSNs in [3] as reproducible in single subject ICA runs.

We compared the reproducible RSNs from the single subject RAICAR-N analysis to the group RSN maps reported in literature [8]. Please see Figure 9.

To summarize, when single subject ICA runs are combined across subjects:

- We are able to declare 4 “standard” RSNs as significantly reproducible at a 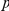-value 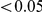.
- There are 2 other “standard” RSNs that achieve a reproducibility 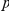-value between 0.05 and 0.06.
- There are 2 other “non-standard” RSNs that are of interest: one achieves a 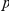-value of 0.0125 and the other achieves a 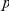-value of 0.05699.

### 3.4 RAICAR-N on random sets of 5 subjects - 50 group ICA runs

**Figure 10:**
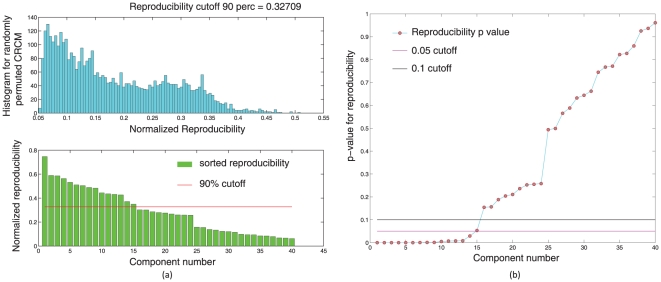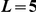 subjects were randomly drawn from the set of 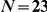 subjects and submitted to a temporal concatenation based group ICA. This process was repeated 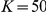 times and the resulting ICA maps were submitted to a RAICAR-N analysis. Figure (a) shows the observed values of normalized reproducibility (bottom) as well as the “null” distribution of normalized reproducibility across 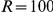 simulations (top). Figure (b) shows the 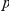-values for each IC along with the 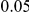 and 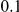 cutoff lines.

To promote diversity across the group ICA runs, as discussed in section 2.5, 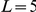 subjects were drawn at random from the group of 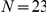 subjects and submitted to a temporal concatenation based group ICA. This process was repeated 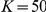 times and the resulting set of 50 group ICA maps were submitted to a RAICAR-N analysis. ICA components were sorted according to their reproducibility and 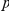-values were computed for each ICA component. Please see Figure 10.

**Figure 11:**
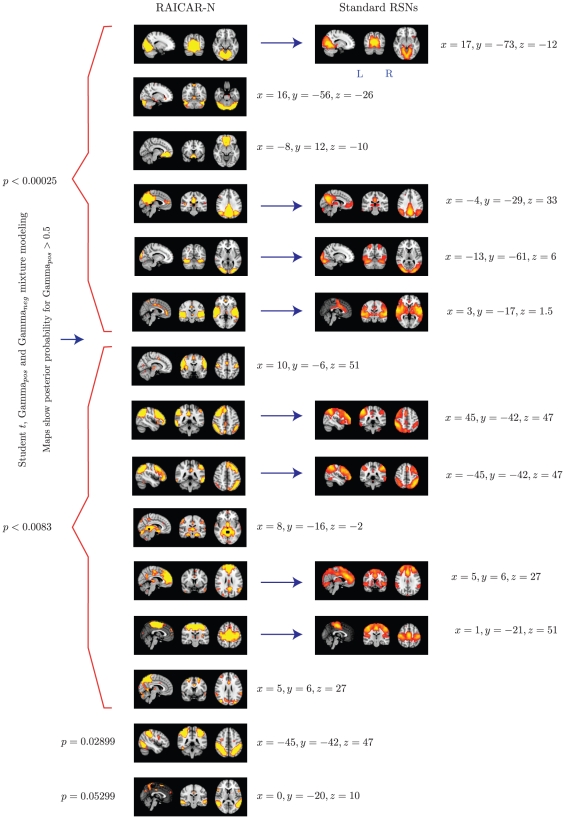
The top 15 “reproducible” ICs from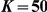 runs of 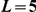 subject group ICA RAICAR-N analysis compared with standard RSN maps reported in literature [8]. We are able to declare 8 “standard” RSNs as significantly reproducible at a 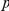-value of 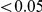. There are 6 other “non-standard” RSNs that can be declared as significantly reproducible at a 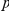-value of 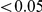 and 1 other “non-standard” RSN that achieves a 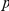-value of 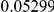.

We compared the reproducible RSNs from the single subject RAICAR-N analysis to the RSN maps reported in literature [8]. Please see Figure 11.

In summary, when 50 random 5 subject group ICA runs (from a population of 23 subjects) are combined using RAICAR-N:

- We are able to declare 8 “standard” RSNs as significantly reproducible at a 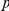-value 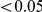.
- There are 6 other “non-standard” RSNs that can be declared as significantly reproducible at a 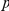-value 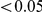.
- There is 1 other “non-standard” RSN that achieves a 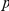-value of 0.05299.

### 3.5 RAICAR-N on random sets of 5 subjects - 100 group ICA runs

**Figure 12:**
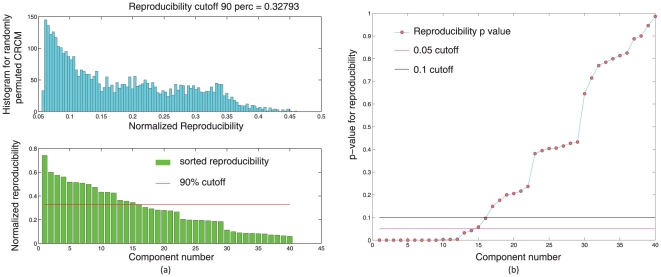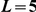 subjects were randomly drawn from the set of 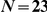 subjects and submitted to a temporal concatenation based group ICA. This process was repeated 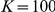 times and the resulting ICA maps were submitted to a RAICAR-N analysis. Figure (a) shows the observed values of normalized reproducibility (bottom) as well as the “null” distribution of normalized reproducibility across 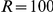 simulations (top). Figure (b) shows the 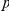-values for each IC along with the 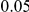 and 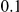 cutoff lines.

To promote diversity across the group ICA runs, as discussed in section 2.5, 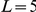 subjects were drawn at random from the group of 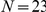 subjects and submitted to a temporal concatenation based group ICA. This process was repeated 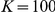 times and the resulting set of 100 group ICA maps were submitted to a RAICAR-N analysis. ICA components were sorted according to their reproducibility and 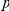-values were computed for each ICA component. Please see Figure 12.

**Figure 13:**
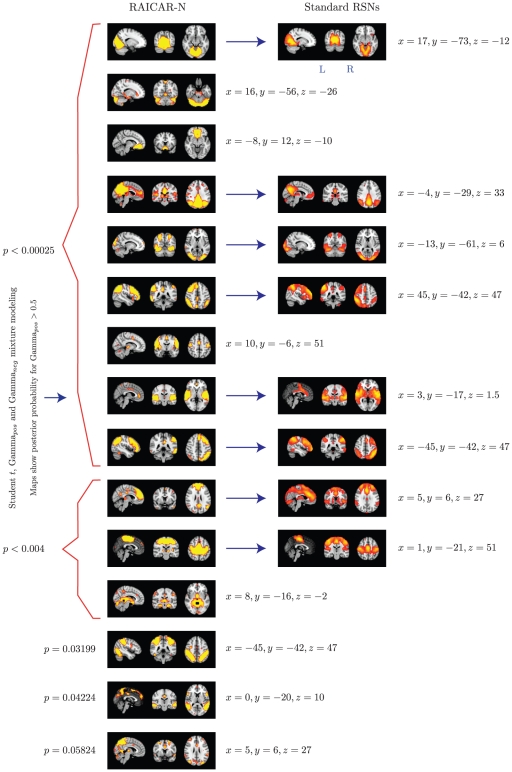
The top 15 “reproducible” ICs from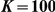 runs of 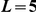 subject group ICA RAICAR-N analysis compared with standard RSN maps reported in literature [8]. We are able to declare 8 “standard” RSNs as significantly reproducible at a 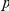-value of 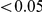. There are 6 other “non-standard” RSNs that can be declared as significantly reproducible at a 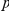-value of 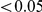 and 1 other “non-standard” RSN that achieves a 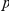-value of 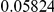.

We compared the reproducible RSNs from the single subject RAICAR-N analysis to the RSN maps reported in literature [8]. Please see Figure 13.

In summary, when 100 random 5 subject group ICA runs (from a population of 23 subjects) are combined using RAICAR-N:

- We are able to declare 8 “standard” RSNs as significantly reproducible at a 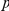-value 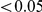.
- There are 6 other “non-standard” RSNs that can be declared as significantly reproducible at a 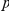-value 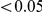.
- There is 1 other “non-standard” RSN that achieves a 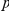-value of 0.05824.

## Discussion

As discussed in section 2.1.2, in the noisy linear ICA model with isotropic diagonal Gaussian noise co-variance, for a given true model order, the mixing matrix and the source distributions are identifiable upto permutation and scaling. However, as pointed out in section 2.1.3, various factors prevent the convergence of ICA algorithms to unique IC estimates. These factors include ICA model not being the true data generating model, approximations to mutual information used in ICA algorithms, multiple local minima in ICA contrast functions, confounding Gaussian noise as well as variability due to model order over-estimation. A practical implication of these factors is that ICA algorithms converge to different IC estimates depending on how they are initialized and on the specific data used as input to ICA. Hence, there is a need for a rigorous assessment of reproducibility or generalizability of IC estimates. A set of reproducible ICs can then be used as ICA based characteristics of a particular group of subjects.

We proposed an extension to the original RAICAR algorithm for reproducibility assessment of ICs within or across subjects (Figure 7). The modified algorithm called RAICAR-N builds up a “null” distribution of normalized reproducibility values under a random assignment of observed ICs across the 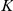 runs. This “null” distribution is used to compute reproducibility 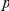-values for each observed matched component from RAICAR. An objective cutoff such as 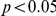 can be used to detect “significantly reproducible” components. This avoids subjective user decisions such as selection of the number of clusters in ICASSO or the reproducibility cutoff in RAICAR or a cutoff on intra cluster distance in sogICA.

### 4.1 Results for publicly available rsfMRI data

We applied RAICAR-N to publicly available 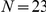 subject rsfMRI data from http://www.nitrc.org/. We analyzed the data in 2 different ways:

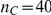
 ICs were extracted for each of the 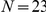 subjects. The 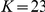 single subject ICA runs were subjected to a RAICAR-N analysis (after registration to standard space).

- In single subject ICA based RAICAR-N analysis (Figures 8, 9), we are able to declare 6 out of the 8 ICs reported in [8] (which used group ICA) as “reproducible” (4 ICs have 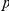-values 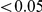 and 2 ICs have 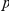-values 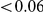). This is consistent with the 5 reproducible RSNs reported in [32] using single subject ICA analysis.

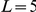
 subjects were randomly drawn from 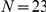 subjects to create one group of subjects which was subjected to a group ICA analysis in which 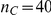 components were extracted. This process was repeated 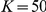 or 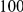 times and the resulting group ICA runs were subjected to a RAICAR-N analysis.

- In group ICA based RAICAR-N analysis (Figures 10, 11, 12, and 13), we are able to declare all 8 components reported in [8] as “reproducible” (at 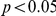). Some of the ICs detected as “reproducible” in the group ICA based RAICAR-N on human rsfMRI data are not shown in [8] but do appear in the more recent paper [31]. RAICAR-N results for 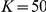 are almost identical to those for 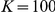 suggesting that 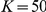 runs of group ICA are sufficient for a RAICAR-N reproducibility analysis.

### 4.2 Single subject ICA vs Group ICA

Based on our results, it appears that single subject ICA maps are less reproducible compared to group ICA maps as illustrated in Figures 8 and 10. A single subject ICA based analysis is more resistant to subject specific artifacts. On the other hand, a group ICA based analysis makes the strong assumption that ICs are spatially identical across subjects. If this assumption is true, group ICA takes advantage of temporal concatenation to constrain the ICs spatially across subjects thereby reducing their variance. Hence, when there are no gross artifacts in individual rsfMRI data sets, group ICA is expected to be more sensitive for reproducible IC detection. As seen in Figures 9 and 11, our results agree with this proposition. All ICs declared as “reproducible” in the single subject based RAICAR-N analysis continue to remain “reproducible” in the group ICA based RAICAR-N analysis.

### 4.3 How should subjects be grouped for group ICA?

This raises the question of how the subjects should be grouped together for individual group ICA runs in preparation for RAICAR-N. If all 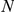 subjects are used in all group ICA analyses then there is no diversity in the individual group ICA runs. In this case, a RAICAR-N analysis will capture algorithmic variability due to non-convexity of ICA objective function but not dataset variability. Hence, our conclusions might not be generalizable to a different set of 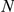 subjects.

Another option is to randomly select 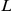 subjects out of 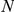 for each group ICA run and submit the resulting 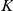 group ICA runs to RAICAR-N. In this case, we will account for both algorithmic and data set variability via a RAICAR-N analysis. In other words, we will be able to determine those ICs that are “reproducible” across different sets of 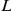 subjects and across multiple ICA runs. A key question is: How should we choose 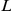 and 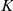
? In section 2.5, we proposed a simple method to determine the number of subjects 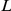 to be used in a single group ICA run out of the 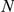 subjects - the key idea is to form groups with enough “diversity”. Multiple such group ICA runs can then be submitted to a RAICAR-N analysis for reproducibility assessment. Clearly, the larger the value of 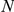, the larger the value of 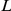. Hence, increasing the number of subjects 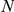 in a study will allow us to make conclusions that are generalizable to a larger set of 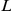 subjects. Also, conclusions generalizable to 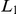 subjects are expected to hold for 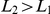 subjects but not vice versa.

### 4.4 RAICAR-N for group comparisons of *reproducible* ICs

In the present work, our focus was on enabling the selection of reproducible ICs for a given single group of subjects. However, RAICAR-N can be extended for between group analysis of reproducible components as well. Before we describe how to do so, it is useful to discuss other approaches for group analysis of RSNs described in **Appendix S1**. Suppose we have two groups of subjects 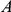 and 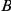.

#### 4.4.1 Discussion of single group ICA based approaches

1. Subject specific maps corresponding to group ICA maps derived using ICA back projection or dual regression are not true ICs, i.e., they are not solutions to an ICA problem.

2. These approaches do not account for either the algorithmic or the data set variability of an ICA decomposition. The single group ICA decomposition will contain both reproducible and non-reproducible ICs, but there is no systematic way to differentiate between the two.

3. Both dual regression and ICA back projection using data derived IC templates are circular analyses. First, group ICA using **all** data is used to derive template IC maps or template time courses. Next least-squares based ICA back projection or dual regression using a subset of the same data is used to derive subject specific maps and time courses corresponding to each IC. Thus model 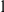 (group ICA) on data 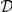 is used to learn an assumption 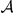 (template IC maps or template time courses) that is then used to fit model 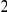 (dual regression or ICA back projection) on a subset of the **same** data 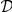. This is circular analysis [33], .

It is easy to avoid circular analysis in a dual regression approach via cross-validation. For example, one can split the groups 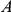 and 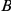 into two random parts, a “training” set and a “test” set. First, the “training” set can be used to derive template IC maps using group ICA. Next, the “training” set based template IC maps can be used as spatial regressors for dual regression on the “test” set. Alternatively, the template ICs for dual regression can also come from a separate ICA decomposition on a independent data set unrelated to groups 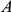 and 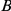 such as human rsfMRI data. This train/test approach cleanly avoids the circular analysis problem. It is not clear how to use cross-validation for an ICA back projection approach since template time courses cannot be assumed to remain the same across ICA decompositions.

4. Subject specific structured noise is quite variable in terms of its spatial structure. Hence, a group ICA analysis cannot easily model or account for subject specific structured noise via group level ICs. Consequently, subject specific spatial maps in ICA back projection or dual regression will have a noise component that is purely driven by the amount of structured noise in individual subjects. On the other hand, a single subject ICA based analysis can accurately model subject specific structured noise via single subject ICs.

#### 4.4.2 Discussion of multiple ICA run approaches

1. [35] report that using different sets of template ICs in template based methods using spatial correlation such as [36] can result in the selection of different ICs in individual ICA runs. This is not surprising since IC correspondence derived from template based methods does depend on the particular template used. This is similar to a seed based correlation analysis being dependent on the particular seed ROI used. It is worth noting that template free approaches such as sogICA and RAICAR do not rely on any template.

2. [22] state that individual runs across subjects (or groups of subjects) can be quite variable in terms of the spatial structure of the estimated ICs. For example [22], point out that an IC might be apparently split into two sub-components in some subjects but not others. The real problem is that the same model order could lead to over-fitting in some subjects (or groups of subjects) but not in others. Hence, the observed differences in a group comparison might be biased by the unknown difference in the amount of over-fitting across groups 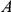 and 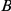.

As described in 2.1.3, over-fitting can lead to the phenomenon of component “splitting” in ICA. This is not limited to single subject ICA but can also occur in group ICA. For instance [35], report the “default mode” network as split into three sub networks using group ICA and note that component “splitting” can also reflect functional segregation or hierarchy within a particular IC and is not necessarily a consequence of model order overestimation in every case.

Over-fitting can be correctly accounted for by a reproducibility analysis. This is because we expect the real and stable non-Gaussian sources to be reproducible across multiple ICA runs (algorithmic variability) and across different subjects or groups of subjects (data set variability).

If we want the results of a between group ICA analysis to be generalizable to an independent group of subjects then we must account for both the algorithmic and data variability of ICA. We propose to modify RAICAR-N for enabling between group comparisons of “reproducible” ICs as follows:

1. Enter multiple within and across subject (or within and across sets of subjects) ICA runs for groups 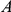 and 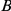 into a RAICAR analysis. Perform the RAICAR component matching process across groups 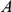 and 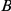.
2. Use RAICAR-N to compute reproducibility 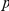-values separately for group 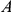 and 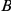 for each matched component across groups 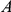 and 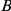.
3. Only ICs that are *separately* reproducible in both groups 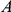 and 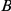 and that are *maximally* similar to each other are used for between group comparisons.

### 4.5 Limitations of a RAICAR-N analysis

In this work, we focussed on developing an objective method for reproducible IC detection across multiple ICA runs. However, our approach has some limitations that are worth mentioning:

1. RAICAR-N gives no indication to the extent of biological relevance of a reproducible IC. For example, an artifactual component consistently appearing across fMRI runs could be highly reproducible and yet non-interesting.
2. The 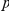-values in RAICAR-N can be conservative. Certain borderline reproducible ICs may not achieve statistical significance in RAICAR-N due to the differences in the amount of structured noise in individual ICA decompositions. One approach to increase the sensitivity of RAICAR-N is to denoise the individual fMRI runs via ICA and remove gross artifacts prior to a RAICAR-N analysis.
3. RAICAR-N does not allow us to *relate* the reproducible ICs to each other. These relationships might be important for the identification of functionally related brain networks. A recent paper [37] proposes to use the mutual information between spatial ICs as a similarity measure for agglomerative heirarchical clustering of ICs. The number of IC clusters is decided using the well-known ANOVA based approach developed in [38]. Each IC cluster can then be thought of as a functionally related brain network.

RAICAR-N is useful for objective and non-parametric reproducibility assessment but does not attempt to relate the ICs to each other. On the other hand, the work by [37] uses a subjective definition of “qualified clusters” and a hard cutoff on the “quality index” to identify the *single most stable run* of ICA for IC clustering. RAICAR-N and the work by [37] have different primary objectives (IC reproducibility vs. IC clustering) and it appears that the benefits of both can be realized by feeding the reproducible ICs from RAICAR-N into the algorithm of [37].

To summarize, a RAICAR-N analysis:

- can be applied for “reproducible” component detection either within or across subjects in any component based analysis - not necessarily ICA. For instance, a set of PCA maps across subjects can be submitted to a RAICAR-N analysis.
- is simple to implement and accounts for both algorithmic and data set variability of an ICA decomposition.
- avoids any user decisions except the final 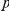-value cutoff which can be objectively pre-set at standard values such as 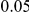.
- can be extended to enable comparisons of reproducible ICs between groups 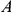 and 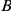.

Multiple group ICA runs using groups of subjects with enough “diversity” can be used to account for the run-to-run variability in ICA algorithms both due to the non-convex ICA objective function as well as across subjects data variability. These group ICA runs can be subjected to a RAICAR-N “reproducibility” analysis. RAICAR-N enables the objective detection of “reproducible components” in any component based analysis of fMRI data such as ICA and can also be used for a between group comparison of “reproducible” ICs.

## Supporting Information

Appendix S1 **Background on group comparison of ICA results.**
(TEX)
